# The proteinase-activated receptor-2 mediates protective immunity in early experimental leishmaniasis

**DOI:** 10.1080/22221751.2026.2725993

**Published:** 2026-09-04

**Authors:** Yvonne Kusche, Niels Münck, Linda Nemetschke, Kirsten Roebrock, Lena Fischer-Riepe, Eva Nattkemper, Kerstin Vischedyk, Martin Steinhoff, Johannes Roth, Cord Sunderkötter, Jan Ehrchen

**Affiliations:** a Department of Dermatology, University of Münster, Münster, Germany; b Institute of Immunology, University of Münster, Münster, Germany; c Department of Dermatology, University of Halle, Halle, Germany; d Department of Dermatology and Venereology, Director of Dermatology Institute, Director of Translational Research Institute (TRI), Hamad Medical Corporation (HMC), Doha, Qatar; e School of Medicine, Weill Cornell University-Qatar; School of Life Sciences, Hamad-bin-Khalifa University; School of Medicine, Qatar University, Doha, Qatar; f Department of Dermatology, Weill Cornell University, New York, NY, USA; g MSB Medical School Berlin, University of Health and Medicine, Berlin, Germany

**Keywords:** Th1/Th2 differentiation, *Leishmania major*, proteinase-activated receptor-2 (PAR2), coagulation factor X (FX and F10 for protein), cytokines, immune microenvironment

## Abstract

Resistance to *Leishmania (L.) major* depends on the development of a *L. major*-specific Th1 response, while Th2 differentiation results in susceptibility. We previously showed that the early microenvironment of infected skin delivers important signals for T cell differentiation. We found increased expression levels of coagulation factor X (F10 and FX for protein, respectively) 16 h after infection in the skin of resistant mice compared to susceptible mice. Activated FX is a ligand of proteinase-activated receptor-2 (PAR2), a modulator of inflammatory responses. To assess the role of PAR2, we analysed the course of *L. major* infection in PAR2-deficient (PAR2^^−^/^−^^) mice on a resistant C57BL/6 background. PAR2^^−^/^−^^ mice developed significantly larger lesions and harboured more parasites in footpads and draining lymph nodes compared to wild-type mice. In addition, their antigen-specific T cell response was shifted towards Th2. Conversely, early treatment of susceptible BALB/c mice with a PAR2-agonist reduced parasite loads in footpads and spleens and shifted the T cell response towards Th1. This was accompanied by significantly higher expression of the Th1-promoting cytokines IL-6, IL-12, and TNFα in the infected skin. We conclude that PAR2 activation favours Th1-differentiation and resistance in experimental leishmaniasis due to an altered initial microenvironment with increased expression of Th1-promoting cytokines in the infected skin.

## Introduction

T-helper (Th) cell differentiation and proliferation have a major impact on the course of diverse infections and inflammatory diseases. Th1 and Th17 subsets dominate in bacterial infections and, when dysregulated, in chronic inflammatory and certain autoimmune diseases. A prevalence of Th2 cells, on the other hand, occurs in helminthic infections and – if dysregulated – allergic or atopic disorders like atopic dermatitis [1–3].

Experimental leishmaniasis is a renowned model system to investigate the mechanisms underlying Th1 and Th2 cell differentiation *in vivo*. A Th1 response in subcutaneous infection with the parasite *Leishmania major* (*L. major*) is crucial for protective immunity in genetically resistant strains such as C57BL/6 mice, as it leads to IFN-γ-mediated activation of macrophages and subsequent killing of intracellular parasites. Susceptible BALB/c mice develop a Th2 response dominated by cytokines like IL-4 and IL-13 and succumb to progressive disease (for review see [4–6]).

The decisive events which determine Th1 – or Th2 differentiation occur early after infection, as pharmacological manipulations can change Th1/Th2 differentiation only if applied during the first two days of infection [7–10]. The Th1/Th2 directing mechanisms have long been supposed to occur primarily in the lymph node during antigen presentation [4,11]. In this process, several signals and co-stimulants are required to shift the immune response towards either Th1 or Th2. Some, but not all of them, have been identified with their respective source [12,13]. There is accumulating evidence that the expression of several of these signals and co-stimulants occurs already in the microenvironment of the primarily infected skin (prior to the events in the draining lymph nodes) [4,9,14]. Using whole-genome expression screening, we characterized the early skin microenvironment in genetically resistant and susceptible mice [15]. We indeed detected differentially regulated genes between resistant and susceptible mice in the infected skin during the crucial time of Th1/Th2 differentiation and identified keratinocytes as one major source – and rich in number – for decisive differential gene induction after dermal infection [3]. As such, we found a significantly stronger up-regulation of cytokines and chemokines in resistant C57BL/6 compared to susceptible BALB/c mice. IL-12, IL-1β, IL-6, and IL-4 belong to those genes which were more strongly upregulated in resistant mice. Expression of these cytokines was temporally restricted to the crucial time of Th1/Th2 differentiation.

IL-12 and IL-1β are already known as important early regulators of Th1 differentiation [7,16,17]. Astonishingly, IL-4, known as an important Th2 cytokine later during infection, had been demonstrated to induce a Th1 response when administered to susceptible mice exclusively in the first hours after infection [18]. We identified the tightly regulated presence of IL-4 in the epidermis during a critical early phase. By neutralizing this cytokine locally in resistant mice within the same timeframe, we observed a shift toward a Th2-type immune response. Moreover, we revealed that IL-6 produced locally in the early infected skin favours the development of Th1 cells and ensuing increased resistance to *L. major* [15]*.* Thus, we clearly demonstrated that cytokines produced locally in the infected skin can direct the ensuing differentiation of a Th1 response in experimental leishmaniasis.

In addition to cytokines, we also found an increased induction of coagulation factor X (F10) in the skin of resistant C57BL/6 compared to susceptible BALB/c mice. While the serine proteinase FX is known as an important part of the coagulation cascade, it also activates proteinase-activated receptor 2 (PAR2) [19,20].

PAR2 is a distinctive member of class A G-protein-coupled receptors (GPCRs) comprising four members: PAR1, PAR2, PAR3, and PAR4 [21]. PARs belong to a family of 7-transmembrane receptors which are initiated by a unique mechanism of self-activation. The extracellular part of PARs contains a specific consensus sequence for certain proteases, and the receptor is cleaved at this sequence by its own activating proteases. Cleavage of the receptor protein results in unmasking of an endogenous protein sequence (“tethered ligand”), which binds the ligand-binding part of the receptor and induces receptor-mediated activation of intracellular signalling cascades. PARs are expressed on many leukocytes as well as keratinocytes or endothelial cells in the skin. PAR2 is activated by cleavage by proteases such as kallikrein (KLK-5 and 14), channel-activating protease (Cap1/Prss8) and FX [20]. Activation of PAR2 may exert proinflammatory effects during skin inflammation [22], and can also indirectly influence Th differentiation in autoimmune disorders like experimental autoimmune encephalitis [19,23]. Thus, we speculated that ligand activation of PAR2 produced early during leishmaniasis could influence Th differentiation and the ensuing course of experimental leishmaniasis. Such a regulatory mechanism would then, most likely, also have broader implications for the generation of Th2 responses in other Th2-driven diseases. We revealed that PAR2 signalling influences the early local microenvironment in the infected skin and contributes to Th1-driven protective immunity in experimental leishmaniasis [24].

The aim of this study was to investigate the early local mechanisms that direct Th1 and Th2 cell differentiation in experimental leishmaniasis, with a particular focus on the roles of FX and PAR2 in the skin.

## Material and methods

### Ethics statement

All experiments with mice were performed with the approval of the State Review Board of Nordrhein-Westfalen (Germany) according to the German law for animal welfare (Tierschutzgesetz) § 8; reference number 8.87-50.10.36.08.009 [15].

### Mice

Specific pathogen-free, male and female C57BL/6 and BALB/c mice were purchased from Charles River, Germany, and were housed in micro-isolator cages and given food and water ad libitum.

PAR2 -/- mice were purchased from Jackson (B6.Cg-F2rl1^tm1Mslb^/J strain #004993). Mice were 8–25 weeks of age when used in experiments. All experiments were approved according to the animal welfare laws of the Federal Republic of Germany by the animal welfare authority of the state of North Rhine-Westphalia, filed under reference 84-02.04. 2015.A348 [25].

### Parasites and experimental leishmaniasis

*L. major* (MHOM/IL/81/FE/BNI) parasites were cultivated in Schneider’s Drosophila Medium supplemented with 10% FCS, 2% human urine, 2% glutamine, and 1% Penicillin/Streptomycin at 25°C and 5% CO_2_. Soluble *Leishmania* antigen (sLA) was prepared by 5 repeated freeze-thaw cycles in phosphate-buffered saline (PBS). Cutaneous leishmaniasis was initiated by subcutaneous application of 2 × 10^7^ promastigotes (stationary phase) of *L. major* in 20 µl PBS into the left hind foot. For gene expression studies, the right foot was injected with 20 μl PBS alone and served as an internal control to ensure that gene expression was not caused by the injection stimulus. For treatment with the PAR2 Agonist, 10^−5^M agonist 2-Furoyl-LIGRLO-amide (Bachem, Germany) in 1μl DMSO or solvent control was included in the final volume and injected simultaneously with, 4 and 24 h after *L. major*. For analysing the early phase of leishmaniasis, mice were killed 4 and 16 h after *L. major* injection, after 11 d (early Th response), and 4 or 5 weeks for infection experiments (late Th response, parasite load). Footpad swelling was measured weekly using a calliper, with the uninfected foot serving as control. Specific swelling of the infected foot was assessed by subtracting the diameter of the infected foot from that of the non-infected foot [15].

### Limiting dilution assay (LDA)

Parasite numbers in the foot pad, draining lymph nodes (dLN) and spleen as a parameter for systemic spread were determined 6–8 weeks after infection by limiting dilution assay (LDA) (modified by using *Leishmania)* medium as specified above instead of slant blood agar [8,25]. Briefly, foot skin, spleen, and dLN were removed aseptically and homogenized in the 5 ml *Leishmania* medium. Serial dilutions were carried out in quadruples (100μl culture volume each) using 96-well tissue plates. After culture for 1 week, the highest dilution yielding growth of viable parasites was determined using a phase-contrast microscope.

### Mixed lymphocyte reaction (MLR)

Cytokines from restimulated *L. major*-specific dLN cells were assessed using a mixed lymphocyte reaction. Briefly, the dLN from infected animals were mashed through a cell strainer in PBS, washed, and transferred to uncoated 96-well U-bottom plates in RPMI 1640 containing 2 mM glutamine, 50 µM mercaptoethanol, and 10% FCS at 2 × 10^6^ cells/well. Cells were restimulated with 1 µl sLA prepared by repeated freeze/thaw cycling of 5 × 10^8^ stationary-phase parasites in 1 ml PBS [25]. After 5 days at 37°C and 5% CO_2_, IFNγ, IL-4, and IL-13 secretion were measured by the cytometric bead array (CBA) using FlexSets by BD Biosciences (San Jose, California) according to the manufacturer’s protocol.

### T cell differentiation

CD4+ T cells were isolated from the spleen of BALB/c mice using the CD4+ T cell isolation Kit (Macs, Miltenyi Biotec, Bergisch Gladbach) and differentiated into the different Th subsets using 3 µg/ml anti-CD3/anti-CD28 (Th0 = “naïve”) and additionally 10 ng/ml recombinant murine (rm) IL-12 + 2 µg/ml anti-IL-4 (Th1) or + 10 ng/ml rmIL-4 + 2ug/ml anti-IFNγ (Th2) for 5 days with and without the addition of 5 µM PAR2 agonist 2-Furoyl-LIGRLO-amide (Bachem Switzerland). Afterwards, T cells were stimulated with PMA/Ionomycin for 4 h, and the supernatant was analysed for Th1- and Th2-specific cytokines using the CBA (BD Bioscience, San Jose, CA, USA) according to the manufacturer’s protocol.

### Measurement of cytokines

To determine secreted proteins in infected foot tissue, dissected feet were flushed repeatedly with a total of 500 µl PBS ( =  exudate) and adjusted to equal protein concentrations [15]. Cytokine concentrations were determined using the CBA (BD Bioscience, San Jose, CA, USA) according to the manufacturer’s protocol.

### Murine cell culture

Murine dendritic cells (DCs) were generated from bone marrow cells as described earlier [26]. Briefly, the bone marrow was flushed from the hind legs of BALB/c mice. After erythrocyte lysis, single cells were seeded into uncovered 6 well plates (2 × 10^6^/2 ml) in the DC medium (RPMI supplemented with 10% fetal calf serum (FCS)), 1% penicillin/streptomycin, 1% L-glutamine, 1% non-essential amino acids (NEAA), 50 µM β-mercaptoethanol, 10 ng/ml rmIL-4 and 10 ng/ml rm-Granulocyte-Macrophage Colony-Stimulating Factor (GMCSF) and incubated at 37°C and 5% CO_2_ for 7 days with change of medium every second day. For the generation of murine macrophages, bone marrow cells were plated in uncovered Petri dishes with the 10 ml of DMEM medium with 4.5 g/L glucose supplemented with 20% L929 supernatant (as a source of Macrophage Colony-Stimulating Factor (M-CSF)), 10% FCS, 2% L-glutamine, 1% penicillin/streptomycin and cultivated at 37°C and 5% CO_2_ for 6 days to differentiate into macrophages.

Production of nitric oxide (NO) was measured by photometric measurement of nitrite in supernatants of bone marrow-derived macrophages at 560 nm using a Griess reagent and a nitrite standard curve after 24 h preincubation of 5 × 10^5^ cells/ml with 500U of rmIFNγ followed by further 24 h incubation with *L. major* and/or 100 ng/ml LPS in the presence of 500U/ml rmIFNγ with and without addition of the 5 µM PAR2 agonist.

### Transfer experiments

To determine how the PAR2 agonist-altered cutaneous microenvironment affects antigen-presenting cells (APCs), we first infected BALB/c mice with 2 × 10^7^ *L. major* parasites with and without 10^−5^M PAR2 agonist for 16 h. We then flushed the feet with 1 ml of PBS and transferred this exudate (containing the locally secreted cytokines) to murine DCs in 1 ml of the culture medium. As a control, 5 µM of the PAR2 agonist was added directly into the DC medium. After incubation for 6 h, DCs were harvested and analysed for Th1-inducing cytokine production using qRT-PCR.

To analyse the capacity of these conditioned DCs to modulate Th differentiation *in vitro*, we incubated these DCs with splenic BALB/c T cells in a 1:10 ratio in a 24-well plate using Th0-polarizing (naïve, only 3 µg/ml anti-CD3/CD28 activation) conditions. After 5 days of co-culture, cells were harvested and analysed for Th1/Th2-inducing genes using qRT-PCR.

To determine a possible role of keratinocytes as target cells of PAR2 signalling, we treated normal human epidermal keratinocytes (NHEKS) for 16 h with or without 5 µM PAR2 agonist. We then transferred the supernatants of the NHEKS onto human APCs (primary human macrophages). As a control, 5 µM PAR2 agonist was added directly to the macrophages. After 16 h, macrophages were harvested and analysed for Th1-inducing cytokine production using qRT-PCR.

### Killing assay

To analyse the phagocytic and killing capacity of macrophages, bone marrow-derived cells from BALB/c mice were differentiated into macrophages, as described in the section “Murine cell culture”.

The killing capacity of bone marrow-derived macrophages from BALB/c was analysed using an adapted LDA. 5 × 10^5^ macrophages were seeded into uncovered 24-well plates, primed for 24 h using rmIFNγ (500U/ml) prior to incubation with *L. major,* with or without 5 µM PAR2 agonist for an additional 24 h. After washing, macrophages were then re-cultured in *L. major* medium at 25°C, performing serial dilutions. After 5 days, the highest dilution yielding growth of viable parasites was determined using a phase-contrast microscope and living *Leishmania* were counted.

### RNA isolation and quantitative RT – PCR (qRT-PCR)

For RNA extraction from mouse feet, skin was homogenized in RNeasy lysis buffer using a PEQLab Precellys homogenizer for a single run at maximum power and time settings. DCs and human macrophages were directly lysed in RNeasy lysis buffer. Afterwards, RNA was extracted using the Qiagen RNeasy micro kit according to the manufacturer’s instructions. Semi – quantitative RT–PCR was performed as described previously on a Bio-Rad CFX384 Touch Real-Time PCR detection system [25,27]. Expression of selected genes was confirmed by real-time RT–PCR, as described previously; see Table 1 for primer sequence information.

**Table 1.** Primer sequences used for qRT-PCR for murine and human samples.

Murine genes	Sequence
*GAPDH*	CCC ACT CTT CCA CCT TCG ATG
	GTC CAC CAC CCT GTT GCT GTA G
*IL-1β*	TGT CTT GGC CGA GGA CTA AGG
	TGG GCT GGA CTG TTT CTA ATGC
*IL-4*	GAG GAC GTT TGG CAC ATC CA
	CAC GGA TGC GAC AAA AAT CAC
*IL-6*	TGA GAT CTA CTC GGC AAA CCT AGT G
	CTT CGT AGA GAA CAA CAT AAG TCA GAT ACC
*IL-12p40*	CAC CCT TGC CCT CCT AAA CC
	TGG TCC CGT GTG ATG TCT TC
*IL-13*	GCC TGT TAC ACT CAA GGT GAT GTG
	CTG TCA CCA TCT TTA TTT CCG GTT T
*TNFa*	CAC CCT TGC CCT CCT AAA CC
	TGG TCC CGT GTG ATG TCT TC
*IP10 (CXCL10)*	TTC ACC ATG TGC CAT GCC
	GAA CTG ACG AGC CTG AGC TAG G
*Gata3*	CTG GAG GAG GAA CGC TAA TGG
	GAT TTG CTA GAC ATC TTC CGG TTT C
*IFNγ*	TGC TGA TGG GAG GAG ATG TCT AC
	TTT CTT TCA GGG ACA GCC TGT TAC
*Tbet*	CCA CCT GTT GTG GTC CAA GTT C
	CCA AGA CCA CAT CCA CAA ACA TC
*Osteopontin*	TCT GAT GAG ACC GTC ACT GCT AGT AC
	CTC AGT CCA TAA GCC AAG CTA TCA C
Human genes	Sequence
*GAPDH*	GCA AAT TCC ATG GCA CCG T
	GCC CCA CTT GAT TTT GGA GG
*IL-1β*	AACAGGCTGCTCTGGGATTC
	GGTCGGAGATTCGTAGCTGG
*IL-12*	TTC TAA GCC ATT CGC TCC TGC
	CAG AAT AAT TCT TGG CCT CGC A
*TGFβ*	ATG GTG TGT GAG ACG TTG ACT GA
	CGA GAG CCT GTC CAG ATG CT

### RNA in situ hybridization (RISH)

For RISH, the complete coding regions of the genes analysed were amplified by PCR and cloned into pBlueScript (Stratagene, La Jolla, CA). For antisense and sense probes, the plasmid was linearized by appropriate restriction enzymes and T7 or T3 RNA polymerase was used to synthesize digoxigenin-labelled probes [15]. The RISH was performed on 4 mm frozen sections according to a protocol described earlier [28].

### Immunohistochemistry

Immunohistochemical staining was performed as described previously [9] using a rabbit anti-mouse PAR2 and a Ly6C antibody (Abcam, Boston, MA, USA).

### Human cell culture

For the generation of human macrophages, peripheral blood monocytes were obtained from fresh human blood leukocyte reduction chambers of platelet apheresis sets from healthy, voluntary whole blood donations of the University Hospital Münster. Cells were isolated by density gradient centrifugation, as described in [29]. The macrophages were differentiated from human monocytes by culture for 6 days in RPMI medium in the presence of 10% human AB serum.

### Statistical analysis

**Figure 1.**
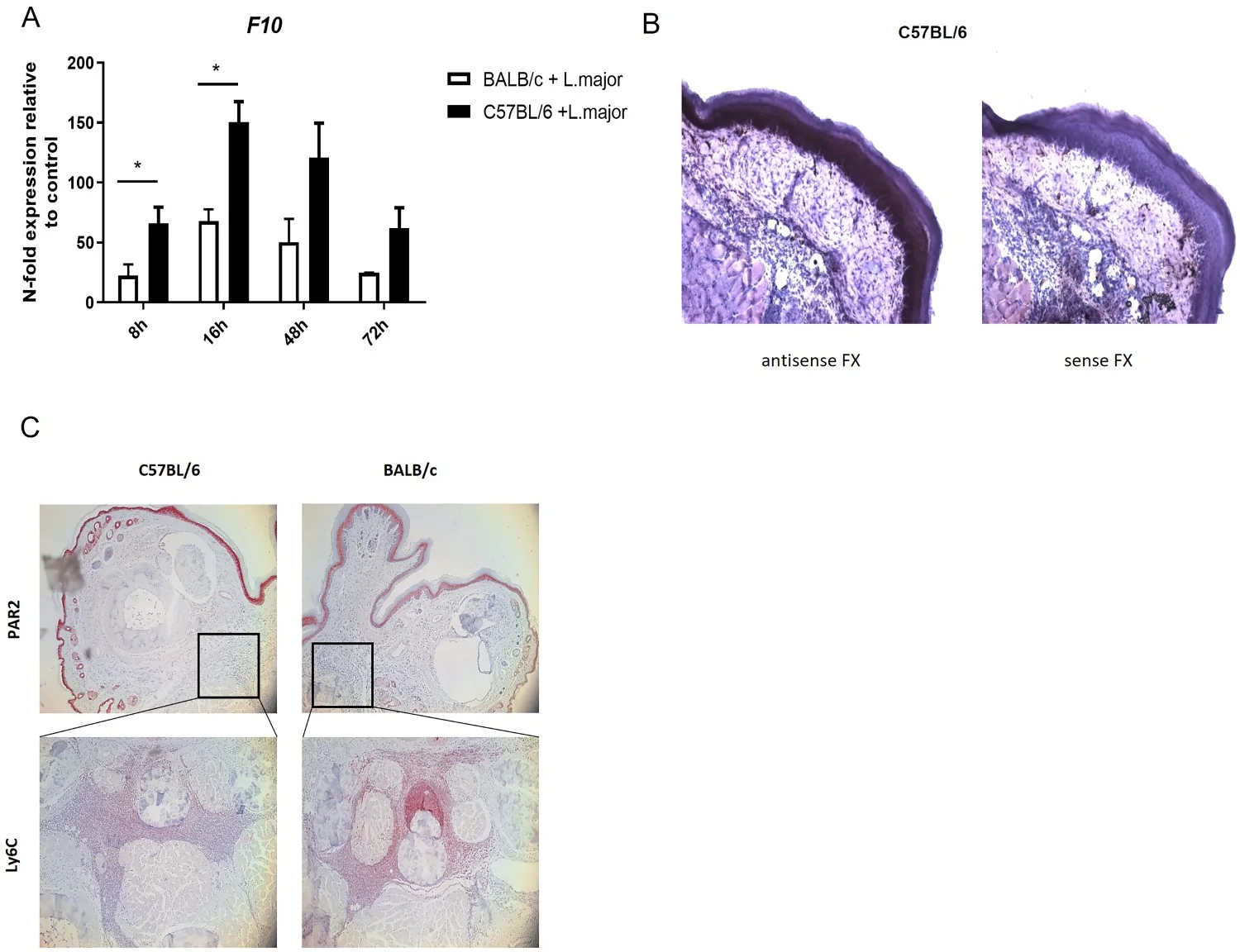
F10 is highly expressed in infected feet of resistant mice. qRT-PCR analysis of *F10* gene expression in *L. major-*infected feet of C57BL/6 and BALB/c mice. The PCR data were normalized to *GAPDH* expression, and mean N-fold regulation and SEM in comparison to PBS-injected control footpads were calculated. Data are means ± SEM from 3 independent experiments with 2 mice per strain and time point (A). *In situ* hybridization was used to analyse the cellular source of FX expression 16 h after *L. major* infection in C57BL/6 feet. Representative images are shown from 3 individual experiments (B). The cellular expression of PAR2 and Ly6C (granulocytes and monocytes/macrophages in the infiltrate) was analysed by immunohistochemistry in paraffin sections of 16 h *L. major-*infected feet of C57BL/6 and BALB/c mice. Representative slides from one mouse per strain are shown; 3 independent experiments with 2 mice were performed (C). * = *P* < 0.05, **  = *P* < 0.01, *** = *P* < 0,001 for differences between C57BL/6 and BALB/c mice.

**Figure 2.**
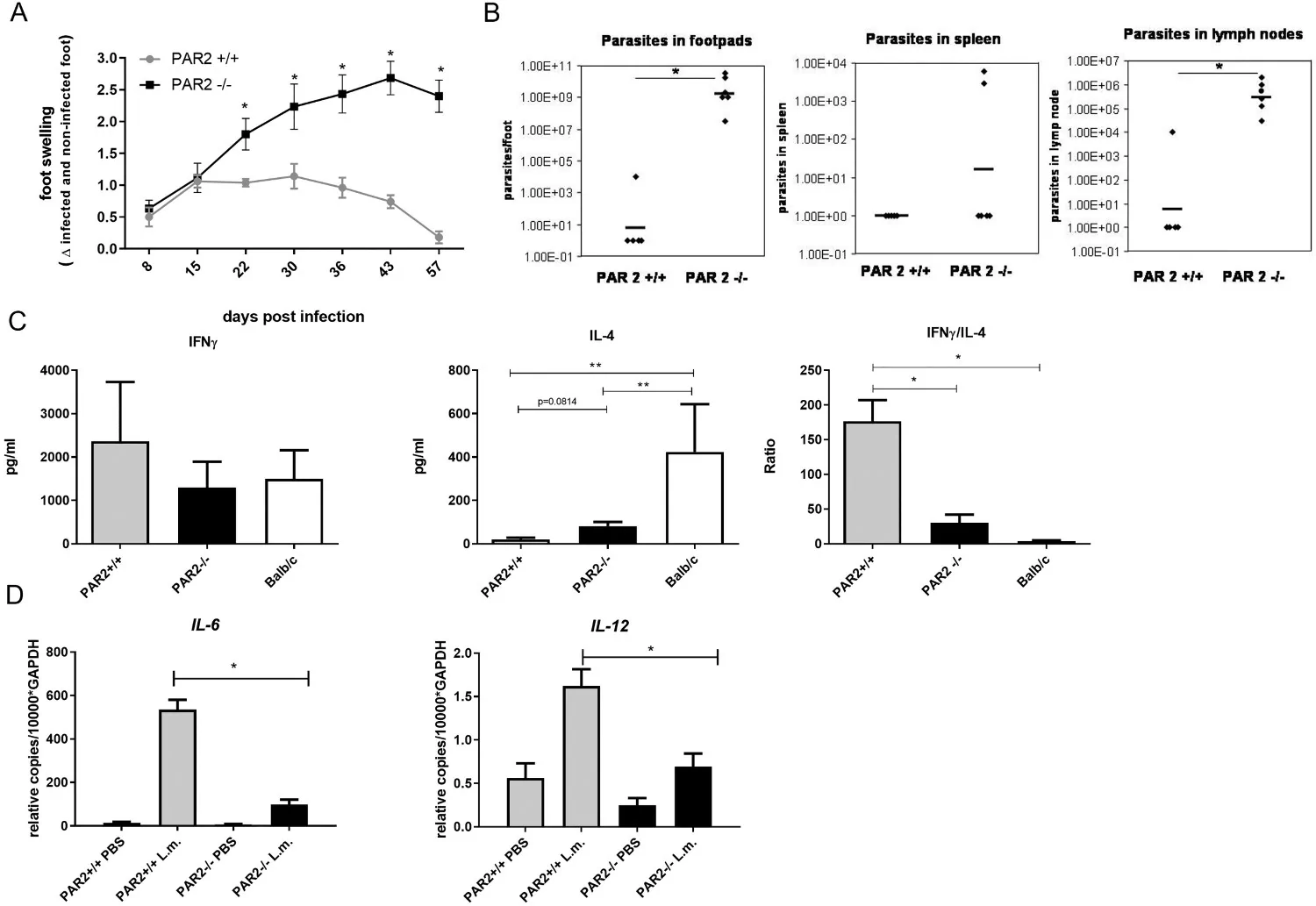
Deterioration of experimental leishmaniasis in PAR2-deficient mice. C57BL/6 (PAR2+/+) and PAR2 KO (PAR2-/-) mice were infected with 2 × 10^7^ *L. major* (6 mice each). The course of the infection was monitored by measuring the foot swelling once a week for 57 days. Differences in footpad swelling between infected and non-infected feet were displayed as means ± SEM from 6 individual mice per group and time point. Data from one representative out of 5 independent experiments with similar results are shown (A). Differences in parasite dissemination were measured by limiting dilution assay of infected feet, spleens, and popliteal lymph node cells after 4 weeks of *L. major* infection. The number of living parasites for single mice (diamonds) and the median for PAR 2+/+ and PAR 2-/- mice (6 mice each) (horizontal bars) are indicated. Data from one representative out of 5 independent experiments are shown (B). Cytokine secretion of dLN cells from PAR2+/+ and PAR2-/- mice after 4 weeks of *L. major* infection. dLN cells were restimulated with soluble *Leishmania* antigen (sLA). IFNγ and IL-4 secretion were measured by CBA after 5 days of cultivation. Data are means ± SEM from 3 independent experiments. * *p* < 0.05; ** *p* < 0.01 (C). mRNA expression of Th1-inducing cytokines IL-6 and IL-12 in foot skin from 4 and 16 h *L. major-*infected C57BL/6 and PAR2-/- mice were determined by qRT-PCR and displayed in relation to the housekeeping gene *GAPDH*. Data are means ± SEM from 3 independent experiments. * *p* < 0.05 (D).

**Figure 3.**
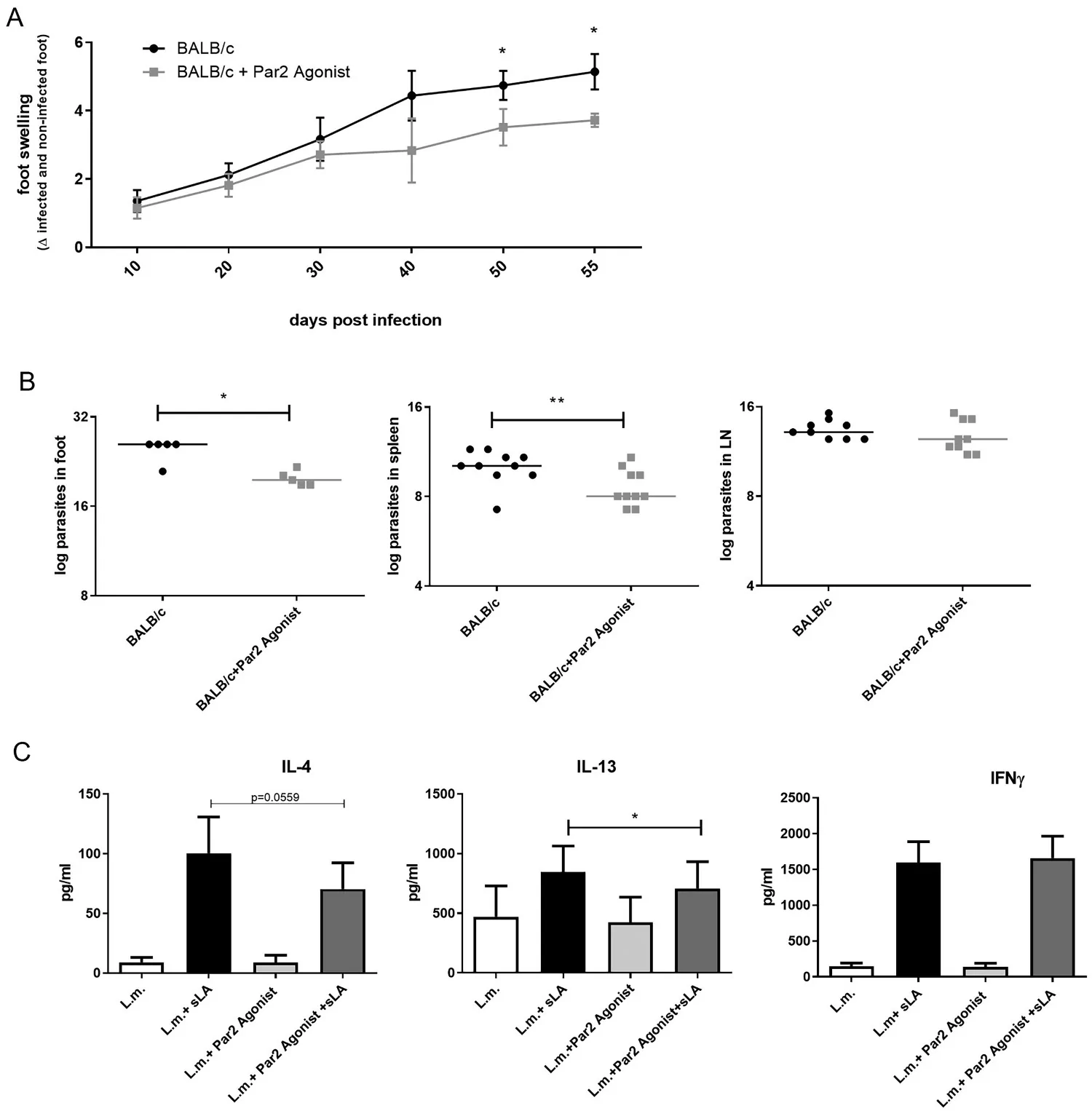
PAR2 Agonist 2-Furoyl-LIGRLO-amide enhances resistance during experimental leishmaniasis in susceptible mice. Treatment of susceptible BALB/c mice with 10^−5^M of PAR2 agonist during inoculation with 2 × 10^7^ *L. major*. 4 and 24 h post-infection (p.i.), mice were additionally treated with a PAR2 agonist. The course of the infection was monitored by measuring the foot swelling once a week over a period of 55 days. The differences in foot swelling between infected and non-infected feet were displayed as means ± SEM from 10 individual mice per group and time point. Data from one representative out of 5 independent experiments with similar results are shown as mean ± SEM. *p < 0.05 vs. control (A). Differences in parasite dissemination were measured by a limiting dilution assay of infected feet, spleens, and dLN cells after 4 weeks of *L. major* infection. The number of living parasites for individual BALB/c animals as controls (black dots) and animals treated with PAR2 agonists (grey squares), as well as their median (horizontal bars), are indicated. Data from one of >3 independent experiments with similar results are shown. The number of biological replicates differed between tissues (footpad: n = 5, spleen: n = 10, draining lymph node: n = 9 per group). * *p* < 0.05; ** *p* < 0.01 (B). Cytokine secretion of dLN cells, which were restimulated with soluble *Leishmania* antigen (sLA) one week after *L. major* infection. After 5 days, IFNγ, IL-4, and IL-13 secretion was measured by CBA. Data are means ± SEM from 3 independent experiments (≥ 3 mice per group) * *p* < 0.05 (C).

**Figure 4.**
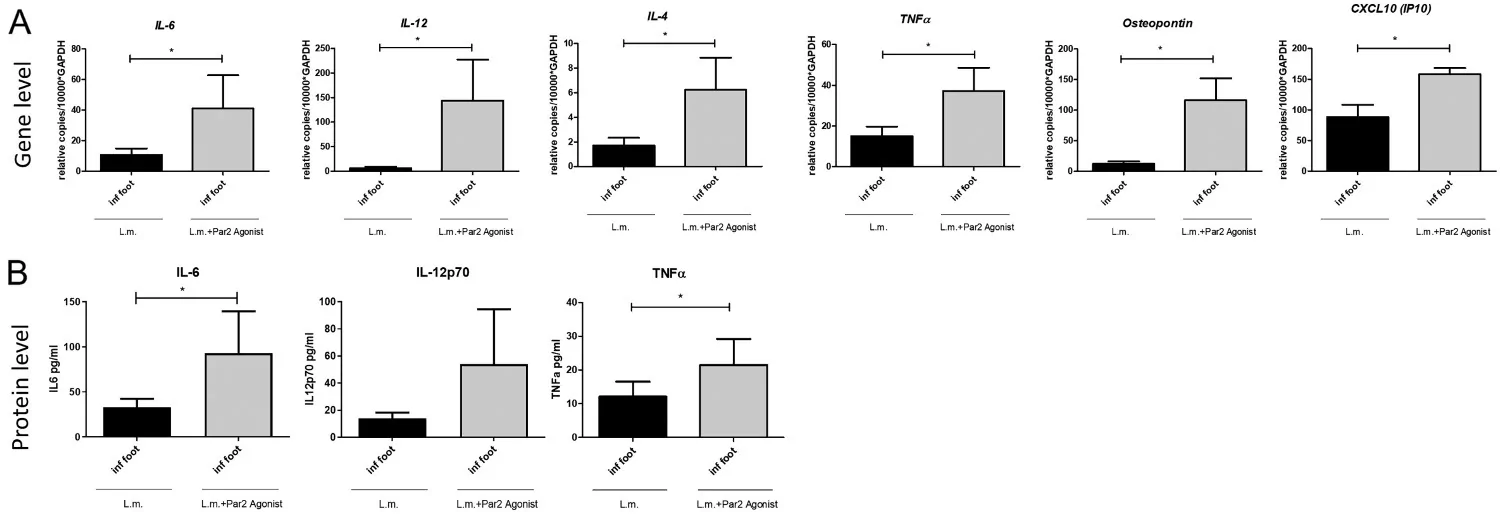
PAR2 Agonist 2-Furoyl-LIGRLO-amide enhances resistance during experimental leishmaniasis in susceptible mice. qRT-PCR (A) and protein (B) analysis of indicated genes in 16 h *L. major-*infected feet of BALB/c mice treated with or without 10^−5^M PAR2 agonist. The qRT-PCR data were normalized to *GAPDH* expression. Proteins were measured in the exudates of infected feet by CBA. Data are means ± SEM from 3 independent experiments. * *p* < 0.05

**Figure 5.**
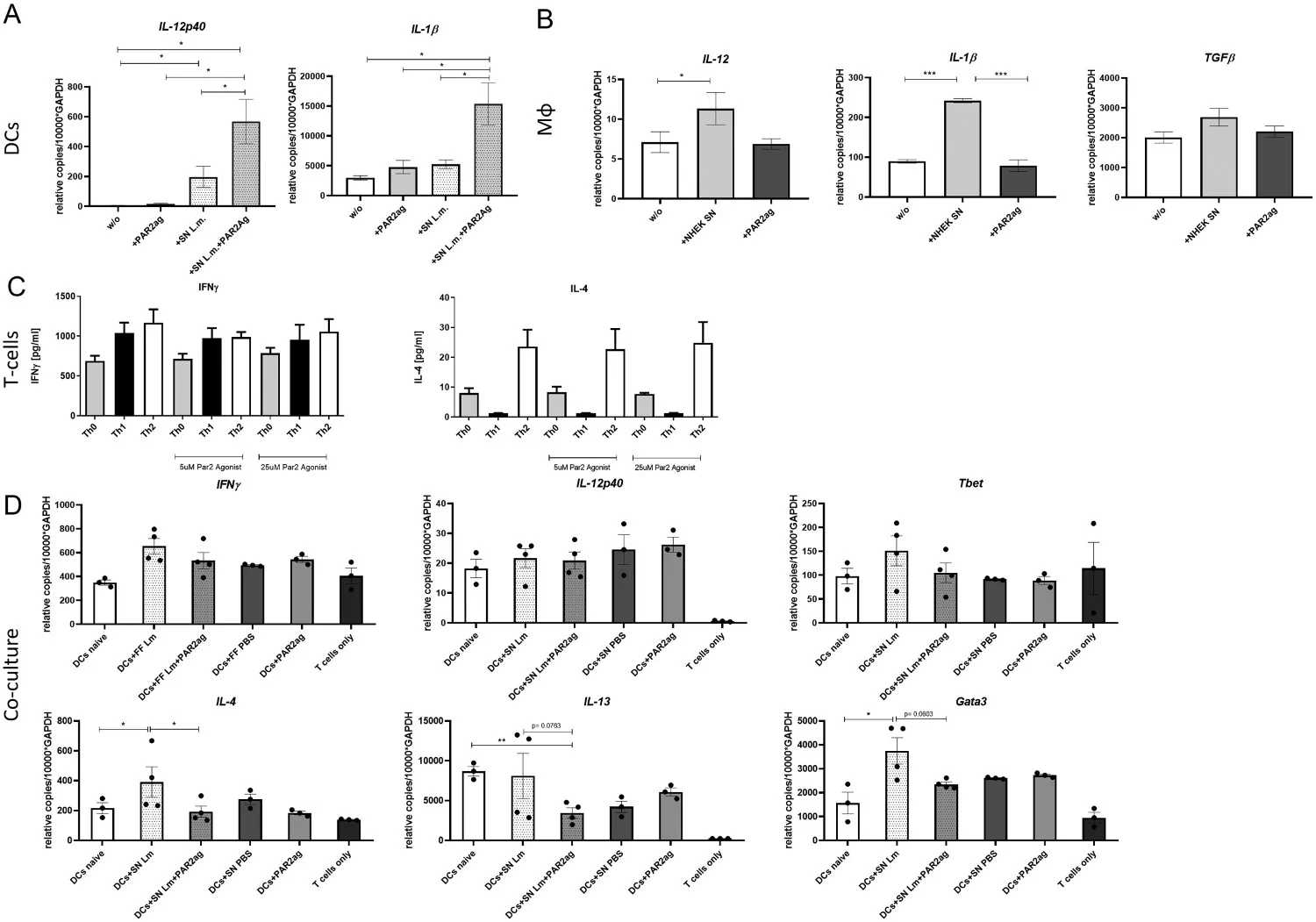
PAR2 signalling modulates the cutaneous cytokine milieu and primes dendritic cells towards a Th1-Inducing phenotype. Murine DCs were incubated with foot exudates (SN) from either BALB/c mice infected with *L. major* (SN L.m.) with or without 10^−5^M PAR2 agonist (SN L.m.+PAR2ag) or PAR2 agonist alone (+PAR2ag) for 6 h. Expression of Th1-inducing cytokines was analysed by qRT-PCR and normalized to *GAPDH* expression. Data are means ± SEM from 3 independent experiments. * *p* < 0.05 (A). Human macrophages were incubated for 16 h with either 5 µM PAR2 agonist alone or supernatant from human keratinocytes (NHEKS SN) treated with or without 5 µM PAR2 agonist for 16 h. Expression of Th1-inducing cytokines was analysed by qRT-PCR and normalized to *GAPDH* expression (B). Data are means ± SEM from 3 independent experiments. * *p* < 0.05, *** *p* < 0.001. Naïve CD4+ T cells from BALB/c mice were differentiated into Th0, Th1, or Th2, as described in Materials and Methods, with or without the addition of 5 µM PAR2 agonist. After 5 days, the supernatants were analysed for secreted IFNy and IL-4 using CBA. Data are means ± SEM from 3 independent experiments (C). Co-culture of BALB/c DCs conditioned for 6 h with or without (DCc naïve) skin exudates collected 16 h after *L. major* infection from BALB/c mice treated with (DCs + SN Lm + Par2ag) or without (DCs + SN Lm) a PAR2 agonist, followed by co-culture with syngeneic CD4⁺ T cells under Th0-polarizing conditions (3 µg/ml anti-CD3/CD28). The exudate from PBS-treated mice (DCs + PBS) as DCs with 5 µM PAR2 agonist alone (DCs + PAR2ag) served as controls. After 5 days, cells were harvested and analysed for Th1/Th2–inducing gene expression using qRT-PCR. Data are means ± SEM from 3 independent experiments. * *p* < 0.05; ** *p* < 0.01 (D).

**Figure 6.**
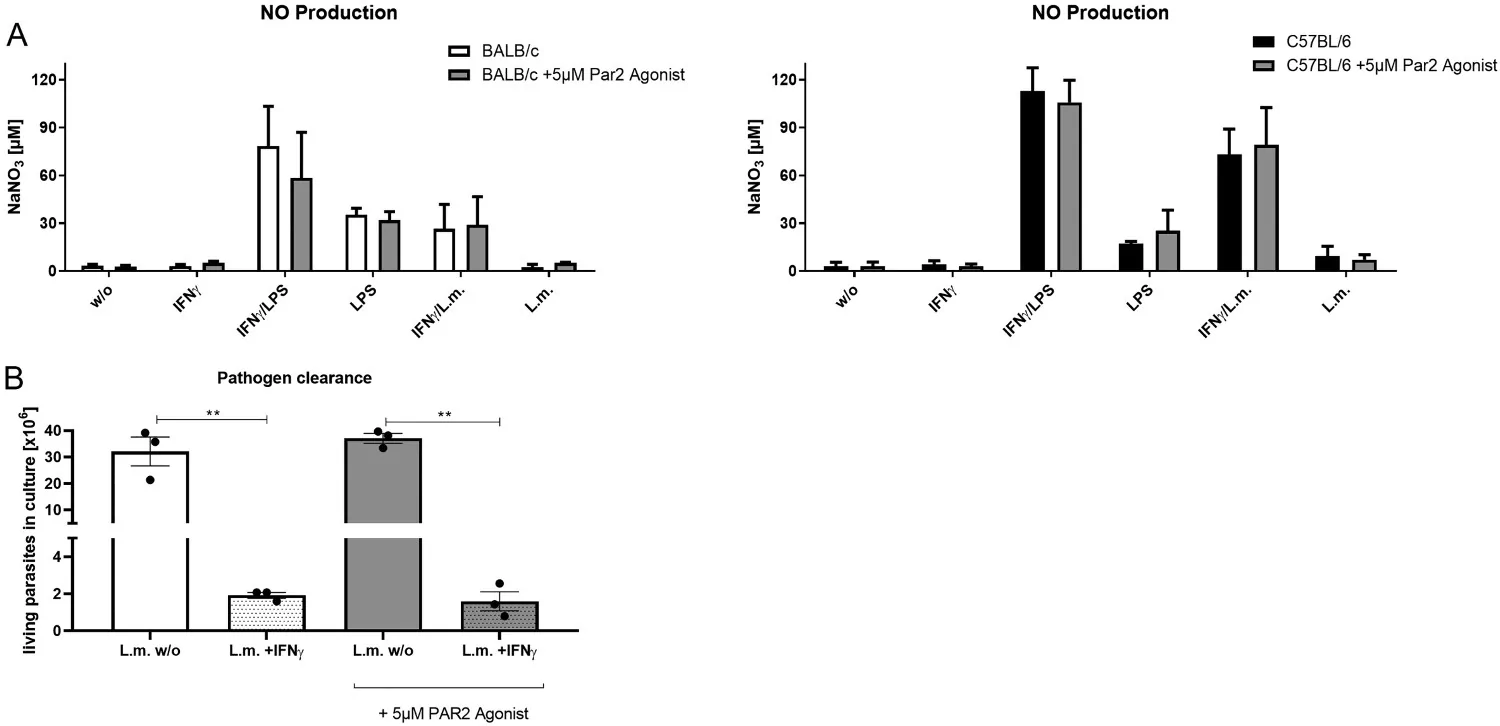
PAR2 signalling did not influence macrophage effector function *in vitro*. Production of nitric oxide (NO) was measured in supernatants of bone marrow-derived macrophages from either BALB/c (left graph) or C57BL/6 (right graph) using Griess reagent. 5 × 10^5^ macrophages were primed using rmIFNγ (500U/ml) 24 h prior to incubation with *L. major* (5:1) and/or (100 ng/ml) LPS for another 24 h (A). The killing capacity of bone marrow-derived macrophages from BALB/c were analysed using an adapted LDA. 5 × 10^5^ macrophages were primed with rmIFNγ 24 h prior to incubation with *L. major* (5:1), additionally with or without 5 µM PAR2 agonist, for another 24 h. Macrophages were re-cultured in the *L. major* medium at 25°C, and serial dilutions and living parasites were counted after 5 days. Living parasites are depicted for each condition. (B). Data are means ± SEM from 3 independent experiments. ** *p* < 0.01.

Data are presented as means ± Standard Error of the Mean (SEM), unless indicated otherwise. A two-tailed Student’s t-test was performed (Figure 1; 2A, C, D; 3A, C; 4, 5, 6) when data were normally distributed (Shapiro-Wilk test). A Mann–Whitney test was performed when data were non-parametrically distributed (Figure 2B and 3B). *p*-values are as follows * < 0.05; ** < 0.01; *** < 0.001.

## Results

### F10 is highly expressed in infected feet of resistant mice

Analysis of differential gene expression by microarray technology in infected skin of resistant versus susceptible mice revealed a 16 h post-infection increase in F10 mRNA in the resistant animals [15]. We now confirmed its regulation and temporal expression by qRT-PCR (Figure 1A). FX expression was temporarily induced in the skin of both susceptible and resistant mice infected with *L. major,* with a peak induction after 16 h and a decline to baseline levels within 2-3 days. In accordance with the microarray results, we revealed a significantly higher expression of F10 in resistant C57BL/6 mice over a period from 8 to 16 h after infection in contrast to susceptible BALB/c mice (Figure 1A).

*In situ* hybridization revealed keratinocytes as the cellular source of FX expression 16 h after infection with *L. major* in C57BL/6 mice (Figure 1B). Since activated FX has been described as a ligand of PAR2, we analysed expression of the latter in infected foot skin of resistant and susceptible mice using immunohistochemistry. Interestingly, we could also localize PAR2 expression to basal keratinocytes in *L. major-*infected resistant and susceptible mice, while PAR2 was not detected in the early leucocyte infiltrate (Figure 1C).

### Deterioration of experimental leishmaniasis in PAR2-deficient mice

To clarify whether FX-PAR2 signalling in general could influence the course of experimental leishmaniasis, we analysed *L. major* infection in C57BL/6 mice with a genetic deficiency of PAR2 (see Materials and Methods). Compared to wild-type mice, PAR2 -/- mice developed significantly larger foot lesions, and we observed an incomplete resolution of infection during the course of our experiment (Figure 2A).

Consistent with the development of higher footpad swelling, PAR2 -/- mice harboured substantially higher numbers of living parasites in footpads and local lymph nodes. In contrast to wild-type mice, we also detected living parasites in the spleen of some PAR2 -/- mice (2/6 compared to 0/5) (Figure 2B), a finding which we and others usually do not observe in the resistant C57BL/6 background of these mice.

To decipher whether the increased susceptibility towards *L. major* infection in PAR2/- mice was associated with a change in the *L. major*-specific T cell response, we analysed parasite-specific production of cytokines by cells of the draining lymph nodes (dLN). The cytokine profile of dLN cells from PAR2^−^/^−^ mice suggested a relative shift towards a Th2 response, resembling that of susceptible BALB/c mice, with lower IFN-γ and higher IL-4 levels and a significantly decreased IFN-γ/IL-4 ratio compared with WT mice (Figure 2C).

Important early sources of cytokines, which influence Th differentiation, are keratinocytes present in the skin. Since we detected PAR2 expression exclusively on keratinocytes in the skin of infected mice, we speculated that FX-PAR2 signalling could have local effects on cutaneous cytokine production. Thus, we analysed early expression of the important Th1-inducing cytokines IL-6 and IL-12 in the skin of wild-type and PAR2-deficient mice at the time point of their peak induction during *L. major* infection [15]. We detected significantly reduced up-regulation of both IL-6 and IL-12 mRNA in PAR2 mutant mice compared to wild-type mice infected with *L. major* (Figure 2D). Taken together, the diminished up-regulation of important Th1-inducing cytokines in the infected skin might favour the Th2 response in PAR2-/- mice.

### The PAR2 Agonist 2-Furoyl-LIGRLO-amide influences experimental leishmaniasis in susceptible mice

Since we saw reduced resistance in mice lacking PAR2 in experimental leishmaniasis, which correlated with a Th2 shift, we wondered if an agonist of PAR2 could re-induce or increase resistance.

The timing of the PAR2 agonist treatment is important, since we detected an increased expression of the PAR2 ligand FX only during the first days of infection and thus in the critical period during which Th1/Th2 differentiation is determined. To specifically manipulate the FX-PAR2 signalling pathway in the early phase of infection, we used locally applicable pharmacological agonists of PAR2. These are small peptides that activate PAR2 without inducing PAR2 proteolysis. These ligands are relatively stable and have been widely used to activate PAR2 *in vivo* [30]. Therefore, we treated susceptible BALB/c mice with the PAR2 agonist 2-Furoyl-LIGRLO-amide only during the first day of Leishmania infection, mimicking the increased period of F10 expression in the skin of resistant mice.

As seen in Figure 3A, mice treated with a PAR2 agonist developed less footpad swelling throughout the time of infection compared to control mice. The effect of treatment on day 1 became significant even in the late phase of infection (day 50 and 55). Analysis of the amounts of parasites in different organs at the end of the experiment (day 50) revealed significantly lower parasite loads in feet and spleens and a trend in draining lymph nodes of BALB/c mice treated with a PAR2 agonist (Figure 3B).

Thus, we could demonstrate that not only the general deficiency of PAR2 influenced experimental leishmaniasis, but also increased PAR2 signalling during the crucial early phase of infection. However, we did not detect a lasting Th1 shift when we analysed the *L. major-*specific cytokine production by re-stimulated dLN cells with sLA (data not shown); early treatment with a PAR2 agonist diminished the genetically dominant Th2 response in BALB/c mice.

*L. major-*specific secretion of IL-13 from draining LN cells one week after infection was significantly reduced in PAR2 agonist treated compared to control mice (Figure 3C). A similar trend was seen for antigen-specific IL-4 secretion; however, this difference did not reach statistical significance (*p* = 0.058). No differences were seen regarding *L. major-*specific IFNγ secretion (Figure 3C). Overall, the data indicate that the early presence of PAR2 agonists in the skin resulted in diminished development of the Th2 response.

We previously showed that there is a significantly higher early cytokine boost in the infected skin of genetically resistant compared to susceptible mice, including many cytokines, which should have the potential to influence Th1/Th2 differentiation.

We, therefore, speculated that, similar to our data from PAR-2 -/- mice, a PAR2 agonist could have affected expression of these cytokines in the infected skin of susceptible mice. We, therefore, analysed expression of such cytokines after early treatment with a PAR2 agonist in *L. major-*infected BALB/c mice. In correspondence with the expression pattern observed in PAR2-/- mice, we could indeed detect higher expression of the Th1-influencing cytokines IL-6 and IL-12 in the skin of BALB/c mice treated with the PAR2 agonist (Figure 4A). Similarly, IL-4 – whose early appearance is pivotal for Th1 differentiation - is upregulated in footpads treated with the PAR2 agonist. Additionally, other cytokines shown to be induced in higher amounts in the skin of resistant mice during early experimental leishmaniasis, such as TNFα and osteopontin, were also induced by PAR2 agonist treatment. Thus, increased PAR2 signalling during the early course of experimental leishmaniasis in susceptible mice resulted in a shift of the skin cytokine milieu towards a milieu detected in resistant mice (Figure 4A).

We detected these changes at both the mRNA and protein levels: We could detect significant upregulation of IL-6 and TNF-α production in footpads of BALB/c mice treated with the PAR2 agonist (Figure 4B). For IL-12, we could see the same trend, but the differences were not statistically significant (Figure 4B).

Thus, while early treatment with the PAR2 agonist may not be sufficient to induce a lasting dominant Th1 response in genetically susceptible BALB/c mice, it does cause an early induction of Th1-inducing cytokines (IL-12, IL-6) in the skin of the PAR2 agonist-treated BALB/c mice in experimental leishmaniasis.

### PAR2 signalling modulates the cutaneous cytokine milieu and primes dendritic cells towards a Th1-inducing phenotype

The PAR2 agonist-altered cutaneous cytokine milieu could prime DCs towards a Th1-inducing phenotype. To support this hypothesis, we incubated bone marrow-derived DCs with exudates derived from the feet of Leishmania-infected BALB/c mice treated either with or without a PAR2 agonist. As a control, DCs were treated with a PAR2 agonist alone. In agreement with a Th1-inducing DC phenotype, we could detect a significantly higher upregulation of the Th1-inducing cytokines IL-12 and IL-1β in DCs treated with foot exudates from PAR2 agonist-treated mice. Treatment of DCs with PAR2 agonist alone did not induce these cytokines (Figure 5A). Thus, the PAR2 agonist-modulated local microenvironment is indeed able to prime DCs towards a Th1-inducing phenotype.

We already demonstrated that keratinocytes are a major source of Th1/Th2-producing cytokines [15]. Since we localized PAR2 to keratinocytes in *L. major-*infected mice, we wanted to test the hypothesis that the activation of PAR2 signalling in keratinocytes could result in the secretion of cytokines which prime APCs towards a Th1-inducing phenotype. As a proof-of-concept experiment, we treated human keratinocytes (NHEKS) with and without a PAR2 agonist and transferred the supernatants to human macrophages. We then analysed macrophage expression of cytokines known to be directly involved in Th1/Th2 differentiation using qRT-PCR.

Notably, the supernatant from PAR2 agonist-treated NHEKs induced production of Th1-inducing cytokines IL-12 and especially IL-1β in macrophages, while production of Th2-inducing TGFβ was not significantly altered. Direct treatment of macrophages with a PAR2 agonist did not result in significant cytokine production. Thus, keratinocytes may represent a relevant target of PAR2 signalling and a source of Th1-promoting cytokine production (Figure 5B).

With respect to Th priming, PAR2 signalling could either directly or indirectly via DCs affect T cell differentiation. Thus, we next investigated if there is a direct priming effect on T cells. To this end, BALB/c T cells were differentiated into the different Th subsets Th0 (“naïve”), Th1 and Th2 for 5 days with and without the addition of different concentrations of the PAR2 agonist. Afterwards, T cells were stimulated with PMA/Ionomycin for 4 h, and the supernatant was analysed for Th1/Th2-specific cytokines. As depicted in Figure 5C, T cells differentiated into Th1, Th2, and Th0 subsets *in vitro,* as shown by the differential profile of secreted Th1 and Th2 cytokines. Addition of a PAR2 agonist during differentiation, however, did not significantly influence the profile of secreted cytokines. Thus, these results do not indicate a direct effect of PAR2 signalling on T cell priming. An indirect effect of PAR2 signalling on T cell differentiation via PAR2 signalling on DCs would be another possibility. However, we did not detect PAR2 by sensitive real-time PCR on DCs (data not shown). Thus, since PAR2 signalling alters the initial cutaneous cytokine milieu in the infected skin and since PAR2 does not directly modify T cells, we conclude that this altered cytokine milieu consecutively affects DCs, the ensuing T cell differentiation and eventually resistance to the parasite.

To further support this hypothesis, we investigated whether DCs conditioned with skin exudates from *L. major*-infected, PAR2 agonist-treated mice could modulate T helper cell differentiation *in vitro*. To address this question, we employed an *in vitro* Th differentiation assay in which the pre-conditioned DCs were co-cultured with naïve CD4^+^ T cells in the presence of 3 µg/ml anti-CD3/CD28 (Th0 (naïve) conditions) (Figure 5D). In agreement with a Th-modulating DC phenotype, the presence of DCs treated with foot exudates from *L. major-*infected, PAR2 agonist-treated mice indeed significantly reduced IL-4 expression from CD4^+^ T cells compared to DCs conditioned without PAR2 agonist exudate. Expression of IL-13 and the master Th2 transcription factor Gata3 were also diminished in CD4^+^ T cells cocultured with DCs conditioned with *L. major* and PAR2 agonist-treated exudates. While these effects are not statistically significant, they are still notable (Figure 5D). Although PAR2 agonist-primed DCs markedly reduced the expression of Th2-associated genes in the T cells, they did not alter the levels of Th1-inducing cytokines (IFN-γ, IL-12p40) or the Th1-defining transcription factor Tbet (Figure 5D).

However, this again supports our hypothesis that PAR2 signalling affects the cutaneous microenvironment, which, in turn, alters DC function and thereby influences the Th1/Th2 response.

### PAR2 signalling did not influence macrophage effector function

Given the effect of PAR2 deficiency and the corresponding effects of PAR2 agonist treatment in the early phase of infection in susceptible BALB/c mice, we wanted to decipher how PAR2 signalling enhances resistance towards *L. major* infection.

Since we observed increased resistance even after treating mice with a PAR2 agonist exclusively on the first day of infection, we assume that PAR2 signalling affects Th1 priming during the crucial early phase of infection.

However, since PAR2 is expressed by macrophages, the major host cells of *Leishmania*, a possible effect of PAR2 signalling on the capacity of macrophages to eliminate *L. major* has to be ruled out. Therefore, we analysed the production of leishmanicidal NO in the supernatants of BALB/c and C57BL/6 macrophages, infected with *Leishmania* for 16 h, and treated with or without a PAR2 agonist. Neither in macrophages of susceptible (BALB/c) nor resistant (C57BL/6) mice strains did we observe an effect of PAR2 ligation on NO production by macrophages (Figure 6A). To evaluate whether pathogen clearance is similarly unaffected,

we performed an *in vitro* infection assay using BALB/c bone marrow-derived macrophages. Therefore, 5 × 10^5^ macrophages were primed using rmIFNγ (500U/ml) 24 h prior to incubation with *L. major* (1:5), additionally with or without a 5 µM PAR2 agonist for another 24 h. Afterwards, macrophages were re-cultured in an *L. major* medium at 25°C, with serial dilutions and viable parasites were counted after 5 days. As shown in Figure 6B, pretreatment of macrophages with IFN-γ significantly reduced the number of viable parasites after 5 days of re-culture, with and without the PAR2 agonist. Consistent with the NO assay results, we did not observe a direct influence of the PAR2 agonist on the killing capacity of macrophages (Figure 6B).

These results indicate that PAR2 signalling does not increase resistance towards *L. major* by virtue of potentiating the killing capacity of macrophages.

## Discussion

We revealed that early expression of F10 in the epidermis and subsequent activation of PAR2 favours Th1 differentiation and resistance in experimental leishmaniasis due to increased expression of Th1-promoting cytokines (IL-6, IL-12, IL-4, TNF-α) in the infected skin. Thus, the FX-PAR2 axis is a decisive component of the early cutaneous Th1/Th2-influencing microenvironment [7,11,15].

In the coagulation pathway, FX cleaves prothrombin into active thrombin, which is a critical step for effective blood coagulation, demonstrated by a severe bleeding phenotype of humans deficient in F10 and the embryonic lethality of F10 null mice [31,32]. In addition, it is known that, besides its role in the coagulation cascade, FX influences immune responses. It signals via PAR2, which represents a distinctive member of G-protein-coupled receptors (GPCRs). Besides PAR1, PAR3, and PAR4, PAR2 is the most highly expressed GPCR on immune cells except for DCs [33–35].

PAR2 activation has proinflammatory effects during skin inflammation [22,36] and can also indirectly influence Th differentiation in autoimmune disorders [30]. The latter is mediated by activation of PAR2 on macrophages and astrocytes resulting in the release of proinflammatory molecules like NO, TNF-α, IL-1β, and IL-6 (the latter e.g. being involved in Th1 differentiation) together with chemokines that, for example, contribute to the recruitment of myelin-reactive T lymphocytes into the central nervous system in a multiple sclerosis or even a Human Immunodeficiency Virus (HIV) model [23,37].

Given the stronger induction of FX in the skin of resistant mice, we wanted to know whether the FX-PAR2 axis is involved in the development of resistance towards *L. major* infection. We were able to detect PAR2 expression in keratinocytes in C57BL/6 and BALB/c mice. This is in line with several studies demonstrating expression of PAR2 in various skin cells, including keratinocytes [20,35,38]. It is also known that PAR2 plays a role in keratinocyte differentiation and proliferation [39,40].

Importantly, we already demonstrated that epidermal keratinocytes are a major source of mediators influencing Th differentiation in the early phase of *L. major* infection [15]. In keratinocytes from resistant mice, we found a strong induction of early Th1-promoting cytokines, such as IL-6, IL-12, and IL-4. Thus, it can be speculated that signalling via PAR2 could influence early cytokine production in keratinocytes and thereby Th1-differentiation in resistant mice.

We verified a decisive contribution of PAR2 signalling in experimental leishmaniasis by detecting a significantly higher number of parasites in infected footpads and draining lymph nodes of PAR2-/- mice. These data supported our hypothesis of a major role of PAR2 in the outcome of *L. major* infection.

Subsequently, we demonstrated a strong Th2 shift in mice deficient in PAR2. Since Th differentiation is determined during the early phase of infection, these data indicate that the major effect of PAR2 signalling on the course of infection could be to promote Th1-differentiation early during infection. PAR2-deficient mice, however, did not allow us to dissect early and possible later effects of the loss of PAR2 signalling on the course of infection.

To support our hypothesis that PAR2 signalling is important for appropriate Th1 differentiation during the early phase of infection, we addressed the question of how early and temporally limited treatment of susceptible mice with PAR2 agonists influences the course of *L. major* infection. Administration of the synthetic PAR2-activating peptide 2-Furoyl-LIGRLO-amide exclusively during the first day of *Leishmania* infection resulted in increased resistance in susceptible mice lasting for weeks, as evidenced by significantly reduced footpad swelling and parasite load in footpads, draining lymph nodes and spleen.

The activation of PAR2 did not induce a full or sustained Th1 shift in mice treated with a PAR2 agonist, but rather a diminished secretion of *L. major-*specific Th2 cytokines, most notably a significant decrease in IL-13. Nevertheless, the biologically relevant IFN-γ/IL-4 ratio was significantly shifted towards a Th1-associated response. This correlates with the fact that PAR2 agonist treatment of susceptible BALB/c mice did not induce complete resistance as seen in C57B/6 mice. Of note, the pharmacological activation produces weaker effects than genetic deletion. This could be due to the epidermal localization of PAR2, which may result in incomplete receptor engagement after dermal injection or due to pharmacokinetic limitations.

Subsequently, a single treatment of susceptible mice with a PAR2 agonist significantly reduced parasite load and dissemination, ameliorated the course of disease and diminished or delayed the development of a strong Th2 response. Given the specific mechanism of PAR2 activation by its ligands resulting in irreversible proteolytic activation and termination of PAR2 signalling by degradation of the receptor [30], it is difficult to exactly mimic the effect of endogenous PAR2 ligands by the use of synthetic PAR2-activating peptides. This could be relevant, since manipulation of the early skin cytokine milieu has to be precisely timed to achieve an alteration of the Th response. As shown for early administration of IL-4, effects may be reversed by extending or shortening the intervention [18]. In line with the diminished Th2 response in lymph nodes, we detected an induction of Th1-inducing cytokines, TNFα, IL-6 and IL-12 in the skin of BALB/c mice treated with a PAR2 agonist. The capacity of PAR2 agonists to induce these cytokines has already been demonstrated in macrophages [23]. Since macrophages may not be the direct target here of the FX/PAR2-axis, further experiments are needed to clarify the mechanistic link from PAR2 activation to TNFα, IL-6, and IL-12 activation.

Thus, PAR2 activation alters the early cytokine milieu in the skin towards the response observed in resistant mice. The altered cytokine milieu in turn could prime DCs towards a Th1-inducing phenotype before they migrate to the local draining lymph nodes and interact with T cells. Supporting this hypothesis, we were able to show that extracts from infected feet treated with a PAR2 agonist induced production of the important Th1-inducing cytokines IL-12 and IL-1β in DCs. The production of IL-12 and IL-1β by DCs is well known to induce Th1 differentiation in experimental leishmaniasis [7,41].

Interestingly, we could indeed show that these DCs, which were pre-conditioned with skin exudates from *L. major*-infected, PAR2 agonist-treated mice, were able to modulate Th-cell differentiation *in vitro*, as demonstrated by the reduced expression of the Th2 cytokines IL-4 and IL-13 as well as the transcription factor Tbet in the subsequent DC–T cell co-culture. To further substantiate these findings, future experiments should quantify the Th1- and Th2-associated cytokines secreted in the co-culture. Additionally, supernatants of keratinocytes treated with a PAR2 agonist *in vitro* also induced the expression of Th1-inducing cytokines IL-1β and IL-12 in macrophages, which is in agreement with keratinocytes being a relevant target of PAR2 signalling and the source of cytokines influencing antigen-presenting cells.

While our data show that PAR2 ligation alters the early cytokine expression in the skin, other effects of PAR2 signalling in experimental leishmaniasis are possible due to the pleiotropic effects of PAR2 on several immune cells [34]. However, we were not able to detect a direct effect of PAR2 ligation on T cell differentiation nor a direct influence of PAR2 activation on macrophages or their killing function.

Thus, we suppose that the main effect of PAR2 activation on experimental leishmaniasis is by shaping the early skin cytokine milieu towards a protective Th1-inducing pattern.

Our data allow the hypothesis that specific targeting of the FX-PAR2 axis may present a potential therapeutic strategy for infections like leishmaniasis and potentially also for other inflammatory skin diseases associated with T cell dysregulation.

## Acknowledgements

The authors thank Ulla Nordhues, Sandra Doths, Claudia Terwesten-Solé, and Gerlinde Schmitz for their outstanding technical support. Yvonne Kusche is a fellow of the InFlame Medical Scientist Kolleg funded by the Else Kröner-Fresenius Foundation.

## References

[1] Stark JM, Tibbitt CA, Coquet JM. The metabolic requirements of Th2 cell differentiation. Front Immunol. 2019;10:2318. doi:10.3389/fimmu.2019.02318. 31611881 PMC6776632

[2] Zhang Y, Zhang Y, Gu W, et al. Th1/Th2 cell’s function in immune system. Dordrecht: Springer; 2014. p. 45–65. doi:10.1007/978-94-017-9487-9_3 25261204

[3] Roebrock K, Sunderkötter C, Münck NA, et al. Epidermal expression of I-TAC (Cxcl11) instructs adaptive Th2-type immunity. FASEB J. 2014;28(4):1724–1734. doi:10.1096/fj.13-233593 24398292

[4] Sacks D, Noben-Trauth N. The immunology of susceptibility and resistance to Leishmania major in mice. Nat Rev Immunol. 2002;2(11):845–858. doi:10.1038/nri933 12415308

[5] Stebut EV. Cutaneous Leishmania infection: progress in pathogenesis research and experimental therapy. Exp Dermatol. 2007;16(4):340–346. doi:10.1111/j.1600-0625.2007.00554.x 17359341

[6] Scott P, Novais FO. Cutaneous leishmaniasis: immune responses in protection and pathogenesis. Nat Rev Immunol. 2016;16(9):581–592. doi:10.1038/NRI.2016.72. 27424773

[7] Von Stebut E, Ehrchen JM, Belkaid Y, et al. Interleukin 1alpha promotes Th1 differentiation and inhibits disease progression in Leishmania major-susceptible BALB/c mice. J Exp Med. 2003;198(2):191–199. doi:10.1084/jem.20030159. 12860932 PMC2194079

[8] Münck NA, Roth J, Sunderkötter C, et al. Aryl hydrocarbon receptor-signaling regulates early leishmania major-induced cytokine expression. Front Immunol. 2019;10:2442. doi:10.3389/fimmu.2019.02442. 31749794 PMC6843081

[9] Sunderkötter C, Kunz M, Steinbrink K, et al. Resistance of mice to experimental leishmaniasis is associated with more rapid appearance of mature macrophages in vitro and in vivo. J Immun. 1993;151(9):4891–4901. doi:10.4049/jimmunol.151.9.4891 8409447

[10] Boitano S, Flynn AN, Schulz SM, et al. Potent agonists of the protease activated receptor2 (PAR2). J Med Chem. 2011;54(5):1308–1313. doi:10.1021/jm1013049 21294569 PMC3069554

[11] Ehrchen JM, Roth J, Roebrock K, et al. The absence of cutaneous lymph nodes results in a Th2 response and increased susceptibility to Leishmania major infection in mice. Infect Immun. 2008;76(9):4241–4250. doi:10.1128/IAI.01714-07. 18625738 PMC2519402

[12] Singh V, Agrewala JN. Regulatory role of pro-Th1 and pro-Th2 cytokines in modulating the activity of Th1 and Th2 cells when B cell and macrophages are used as antigen presenting cells. BMC Immunol. 2006 Aug 7;7(1):1–10. doi:10.1186/1471-2172-7-17/FIGURES/6. 16889674 PMC1550426

[13] Li Y, Li L. Contact dermatitis: classifications and management. Clin Rev Allergy Immunol. 2021;61(3):245–281. doi:10.1007/S12016-021-08875-0. 34264448

[14] Constant SL, Brogdon JL, Piggott DA, et al. Resident lung antigen-presenting cells have the capacity to promote Th2T cell differentiation in situ. J Clin Invest. 2002;110(10):1441–1448. doi:10.1172/JCI16109. 12438442 PMC151814

[15] Ehrchen JM, Roebrock K, Foell D, et al. Keratinocytes determine Th1 immunity during early experimental leishmaniasis. PLoS Pathog. 2010;6(4):e1000871–16. doi:10.1371/journal.ppat.1000871. 20442861 PMC2861693

[16] Zundler S, Neurath MF. Interleukin-12: functional activities and implications for disease. Cytokine Growth Factor Rev. 2015;26(5):559–568. doi:10.1016/j.cytogfr.2015.07.003. 26182974

[17] Arbore G, West EE, Spolski R, et al. T helper 1 immunity requires complement-driven NLRP3 inflammasome activity in CD4^+^ T cells. Science. 2016;352(6292):aad1210. doi:10.1126/science.aad1210. 27313051 PMC5015487

[18] Biedermann T, Zimmermann S, Himmelrich H, et al. IL-4 instructs TH1 responses and resistance to Leishmania major in susceptible BALB/c mice. Nat Immunol. 2001;2(11):1054–1060. doi:10.1038/ni725 11600887

[19] Göbel K, Asaridou CM, Merker M, et al. Plasma kallikrein modulates immune cell trafficking during neuroinflammation via PAR2 and bradykinin release. Proc Natl Acad Sci USA. 2019;116(1):271–276. doi:10.1073/pnas.1810020116. 30559188 PMC6320546

[20] Guenther F, Melzig MF. Protease-activated receptors and their biological role - Focused on skin inflammation. J Pharm Pharmacol. 2015;67(12):1623–1633. doi:10.1111/jphp.12447 26709036

[21] Buddenkotte J, Stroh C, Engels IH, et al. Agonists of proteinase-activated receptor-2 stimulate upregulation of intercellular cell adhesion molecule-1 in primary human keratinocytes via activation of NF-kappa B. J Invest Dermatol. 2005;124(1):38–45. doi:10.1111/j.0022-202X.2004.23539.x 15654951

[22] Seeliger S, Derian CK, Vergnolle N, et al. Proinflammatory role of proteinase-activated receptor-2 in humans and mice during cutaneous inflammation in vivo. FASEB J. 2003;17(13):1871–1885. doi:10.1096/FJ.02-1112COM. . 14519665

[23] Noorbakhsh F, Tsutsui S, Vergnolle N, et al. Proteinase-activated receptor 2 modulates neuroinflammation in experimental autoimmune encephalomyelitis and multiple sclerosis. J Exp Med. 2006;203(2):425–435. doi:10.1084/jem.20052148. 16476770 PMC2118197

[24] Kusche Y, Münck N, Roebrock K, et al. The proteinase activated receptor-2 mediates protective immunity in early experimental Leishmaniasis. Joint Meeting of the German Society for Immunology (DGfI) and the Austrian Society for Allergology and Immunology. European J Immunol. 2022;52:370–370.

[25] Osier F, Uzonna JE. Editorial: regulation of immunity to parasitic infections endemic to Africa. Frontiers Media S.A. 2020;11. doi:10.3389/fimmu.2020.01159. PMC7304307 32595637

[26] Varga G, Balkow S, Wild MK, et al. Active MAC-1 (CD11b/CD18) on DCs inhibits full T-cell activation. Blood 109, 2, 2007, 661–669. doi:10.1182/blood-2005-12-023044 17003381

[27] Ehrchen J, Steinmu L, Barczyk K, et al. Glucocorticoids induce differentiation of a specifically activated, anti-inflammatory subtype of human monocytes. Hematology. 2007;109(3):1265–1274. doi:10.1182/blood-2006-02-001115.The 17018861

[28] Ehrchen J, Heuer H, Sigmund R, et al. Expression and regulation of osteopontin and connective tissue growth factor transcripts in rat anterior pituitary. J Endocrinol. 2001;169(1):87–96. doi:10.1677/joe.0.1690087. 11250650

[29] Peters AF, Kusche Y, Gerdkamp H, et al. UVA1 radiation attenuates pro-inflammatory functions in human monocytes. J Dermatol. 2023;50(1):46–56. doi:10.1111/1346-8138.16600 36184911

[30] Rothmeier AS, Ruf W. Protease-activated receptor 2 signaling in inflammation. Semin Immunopathol. 2012;34(1):133–149. doi:10.1007/S00281-011-0289-1. 21971685

[31] Menegatti M, Peyvandi F. Factor X deficiency. Semin Thromb Hemost. 2009;35(4):407–415. doi:10.1055/S-0029-1225763. 19598069

[32] Veneri D, Giuffrida AC, Bonalumi A, et al. Use of prothrombin complex concentrate for prophylaxis of bleeding in acquired factor X deficiency associated with light-chain amyloidosis. Blood Transfus. 2016 Nov 1;14(6):585–586. doi:10.2450/2016.0335-15. 27416580 PMC5111391

[33] Cocks TM, Fong B, Chow JM, et al. A protective role for protease-activated receptors in the airways. Nature. 1999;398(6723):156–160. doi:10.1038/18223. 10086357

[34] Henehan M, De Benedetto A. Update on protease-activated receptor 2 in cutaneous barrier, differentiation, tumorigenesis and pigmentation, and its role in related dermatologic diseases. Exp Dermatol. 2019;28(8):877–885. doi:10.1111/exd.13936 30972831

[35] Fleischer MI, Röhrig N, Raker VK, et al. Protease- and cell type–specific activation of protease-activated receptor 2 in cutaneous inflammation. J Thromb Haemostasis. 2022;20(12):2823–2836. doi:10.1111/JTH.15894. 36161697

[36] Steinhoff M, Vergnolle N, Young SH, et al. Agonists of proteinase-activated receptor 2 induce inflammation by a neurogenic mechanism. Nat Med. 2000;6(2):151–158. doi:10.1038/72247. 10655102

[37] Sachan V, Lodge R, Mihara K, et al. HIV-induced neuroinflammation: impact of PAR1 and PAR2 processing by furin. Cell Death Differ. 2019;26(10):1942. doi:10.1038/S41418-018-0264-7. 30683917 PMC6748122

[38] Steinhoff M, Corvera CU, Thoma MS, et al. Proteinase-activated receptor-2 in human skin: Tissue distribution and activation of keratinocytes by mast cell tryptase. Exp Dermatol. 1999;8(4):282–294. doi:10.1111/J.1600-0625.1999.TB00383.X 10439226

[39] Rattenholl A, Steinhoff M. Proteinase-activated receptor-2 in the skin: receptor expression, activation and function during health and disease. Drug News Perspect. 2008;21(7):369–381. doi:10.1358/DNP.2008.21.7.1255294. 19259550

[40] Zhao J, Munanairi A, Liu XY, et al. PAR2 mediates itch via TRPV3 signaling in keratinocytes. J Invest Dermatol. 2020 Jan 29;140:1524–1532. 10.1016/j.jid.2020.01.012. 32004565 PMC7387154

[41] Filippi C, Hugues S, Cazareth J, et al. CD4+ t cell polarization in mice is modulated by strain-specific major histocompatibility complex-independent differences within dendritic cells. J Exp Med. 2003;198(2):201–209. doi:10.1084/JEM.20021893. 12860929 PMC2194066

